# Identification of All Dengue Serotypes in Nepal

**DOI:** 10.3201/eid1410.080432

**Published:** 2008-10

**Authors:** Sarala Malla, Garib D. Thakur, Sanjaya K. Shrestha, Manas K. Banjeree, Laxmi B. Thapa, Gyanendra Gongal, Prakash Ghimire, Bishnu P. Upadhyay, Purosotam Gautam, Shyam Khanal, Ananda Nisaluk, Richard G. Jarman, Robert V. Gibbons

**Affiliations:** National Public Health Laboratory, Kathmandu, Nepal (S. Malla, B.P. Upadhyay, S. Khanal); Epidemiology and Disease Control Division, Kathmandu (G.D. Thakur, M.K. Banjeree, L.B. Thapa, P. Gautam); Walter Reed/Armed Forces Research Institute of Medical Sciences, Kathmandu (S.K. Shrestha); WHO Regional Office for South-East Asia, New Delhi, India (G. Gongal); Tribhuvan University, Tribhuvan, Nepal (P. Ghimire); United States Army Medical Command–Armed Forces Research Institute of Medical Sciences, Bangkok, Thailand (A. Nisaluk, R.G. Jarman, R.V. Gibbons)

**Keywords:** dengue, Nepal, Aedes, serotype, PCR, ELISA, letter

**To the Editor:** Nepal is situated on the southern slopes of the Himalayas, surrounded by India on 3 sides and China to the north. Nepal’s altitude ranges from 8,848 m in the Himalayas to 90 m in the Terai, the southern, low, flatland bordering India. Nepal is a disease-endemic area for many vector-borne diseases, including malaria, kala-azar, Japanese encephalitis, and lymphatic filariasis. Because of the porous border between Nepal and India, social, cultural, and economic activities in cross-border areas are common.

Dengue is an emerging disease in Nepal; presumably transmission is moving north from India into the Terai (*1**–**5*). The first report of dengue virus isolation or RNA (serotype 2 with nucleotide homology closest to a dengue virus type 2 isolate from India) was in 2008 involving a Japanese patient returning from Nepal in October 2004 (*5*). Entomologic investigations from the 1980s showed *Aedes albopictus* in the Terai plains, but *Ae. aegypti* has not been previously reported.

After Indian outbreaks now known to include all 4 dengue serotypes (*6*), a team from the Epidemiology and Disease Control Division, Kathmandu, investigated suspected cases of dengue fever during September–October 2006 in Banke, the district bordering Uttar Pradesh, India. The team collected blood samples from persons in Banke and, subsequently, from persons in a number of other districts and sent them to the National Public Health Laboratory in Kathmandu or the Armed Forces Research Institute of Medical Sciences, Bangkok, Thailand for analysis with ELISA, reverse transcription–PCR, (RT-PCR), or both.

Case definitions for dengue fever were adopted based on World Health Organization guidelines (*7*). Blood samples were obtained from patients with an acute febrile illness of 2–7 days’ duration and with >2 of the following manifestations: headache, retro-orbital pain, muscular or joint pain, and rash. If laboratory tests were positive, cases were confirmed. Results were confirmed by ELISA performed at the Armed Forces Research Institute of Medical Sciences as previously described (*8*). Positive results were immunoglobulin (Ig) M >40 units or IgG >100 units. RT-PCR was performed by extracting RNA from 140 μL of each serum sample using QIAGEN Viral RNA Extraction Kit per manufacturer’s instructions (QIAGEN, Germantown, MD, USA). RT-PCR and nested PCR were conducted according to the Lanciotti protocol (*9*) with the following modifications. Reverse transcriptase from avian myeloblastosis virus (Promega, Madison, WI, USA) was used in the first round RT-PCR. The concentrations of the primers used in the RT-PCR and nested reactions were reduced from 50 pmol to 12.5 pmol per reaction, and the number of nested PCR amplification cycles was increased to 25.

Serum specimens were obtained from 70 suspected case-patients from 16 districts from October 13 through December 3, 2006; 25 confirmed cases (13 by ELISA, 10 by RT-PCR, and 2 by both tests) came from 9 districts (Table). The average age was 29 years (range 5–65 years); 80% of the case-patients were men. Three patients had a history of travel to India, but clusters of dengue fever cases reported in October (Banke and Dang districts) indicated local transmission was occurring among patients with no travel history. The Terai districts accounted for 80% of cases. Entomologic collections done indoors and outside at 5 different sites reporting suspected cases identified *Ae. albopictus* and *Ae. aegypti* in all 5 districts.

**Table:** Dengue laboratory test results, National Public Health Laboratory, Nepal, and AFRIMS, Bangkok, 2006*

Patient no.	Age, y	Gender	Residence	Travel history	ELISA	RT-PCR
1	39	M	Kathmandu	Unknown	Positive	DEN-3
2	48	M	Banke	Yes	Negative	DEN-3
3	18	M	Banke	No	Negative	DEN-3
4	20	M	Banke	No	Negative	DEN-3
5	22	M	Banke	No	Negative	DEN-3
6	25	M	Banke	No	Negative	DEN-3
7	25	M	Kathmandu	Unknown	Negative	DEN-1
8	26	F	Kathmandu	Unknown	Positive	DEN-3
9	38	M	Parsa	No	Negative	DEN-4
10	16	M	Dhading	Unknown	Negative	DEN-2
11	25	M	Jhapa	No	Positive	ND
12	37	F	Parsa	Unknown	Positive	ND
13	38	M	Dhading	No	Positive	ND
14	24	M	Banke	No	Positive	ND
15	36	M	Banke	No	Positive	ND
16	22	M	Parsa	Unknown	Positive	ND
17	5	F	Rupandehi	No	Positive	ND
18	13	M	Dang	No	Positive	ND
19	35	F	Parsa	No	Positive	ND
20	20	M	Kathmandu	Yes	Positive	ND
21	40	M	Kapilbastu	No	Positive	ND
22	20	F	Rupandehi	No	Positive	ND
23	42	M	Dang	No	Positive	ND
24	65	M	Banke	Yes	Positive	ND
25	28	M	Dang	No	Positive	ND

*AFRIMS, Armed Forces Research Institute of Medical Sciences; RT-PCR, reverse transcription–PCR; DEN, dengue; ND, not done.

These clinical and laboratory test results confirmed the presence of all 4 dengue serotypes. Notably, patients from the Dang district had no travel history outside the Dang valley. Because *Aedes* spp. have been identified in Dang, the data strongly suggest the existence of an endemic cycle of dengue. Underreporting is expected in the absence of diagnostic facilities at the field level. It is unclear whether the predominance of male patients is indicative of greater outdoor as opposed to indoor transmission. Of note, *Ae. albopictus* has been found in the country since the 1980s; in this study, we found *Ae. aegypti* in Nepal. Men typically wear short-sleeved clothes due to hot and humid conditions and, therefore, are frequently exposed to mosquito bites. However, men may also access the healthcare system more frequently. The ages of case-patients point to a relative lack of dengue immunity among the older population, and this finding is consistent with a new introduction of dengue. Because dengue hemorrhagic fever appears when >1 serotype becomes endemic to an area (*10*), the presence of all 4 serotypes portends the emergence of more severe dengue disease in Nepal.
